# Key Technologies for Progressing Discovery of Microbiome-Based Medicines

**DOI:** 10.3389/fmicb.2021.685935

**Published:** 2021-06-22

**Authors:** Remy B. Young, Vanessa R. Marcelino, Michelle Chonwerawong, Emily L. Gulliver, Samuel C. Forster

**Affiliations:** ^1^Centre for Innate Immunity and Infectious Diseases, Hudson Institute of Medical Research, Clayton, VIC, Australia; ^2^Infection and Immunity Program, Monash Biomedicine Discovery Institute and Department of Microbiology, Monash University, Clayton, VIC, Australia; ^3^Department of Molecular and Translational Sciences, Monash University, Clayton, VIC, Australia

**Keywords:** microbiome, faecal transplant, gastrointestinal disorder, 16S rRNA sequencing, metagenomic sequencing, microbial genomics, bacteriotherapy, live biotherapeutics

## Abstract

A growing number of experimental and computational approaches are illuminating the “microbial dark matter” and uncovering the integral role of commensal microbes in human health. Through this work, it is now clear that the human microbiome presents great potential as a therapeutic target for a plethora of diseases, including inflammatory bowel disease, diabetes and obesity. The development of more efficacious and targeted treatments relies on identification of causal links between the microbiome and disease; with future progress dependent on effective links between state-of-the-art sequencing approaches, computational analyses and experimental assays. We argue determining causation is essential, which can be attained by generating hypotheses using multi-omic functional analyses and validating these hypotheses in complex, biologically relevant experimental models. In this review we discuss existing analysis and validation methods, and propose best-practice approaches required to enable the next phase of microbiome research.

## Introduction

The human microbiome has now been implicated in several pathologies, including inflammatory bowel disease (IBD; Ott et al., 2004), diabetes (Vatanen et al., 2018), and obesity (Cox et al., 2014; de la Cuesta-Zuluaga et al., 2018) and therefore represents a broad-range potential therapeutic target. Evidence suggests microbiome-based interventions such as probiotics, may reduce symptoms of irritable bowel syndrome (IBS; Ford et al., 2014), antibiotic- associated diarrhea (Evans et al., 2016; Blaabjerg et al., 2017), preterm infant mortality rates and necrotizing enterocolitis (Sekhon et al., 2019; Underwood, 2019; Liu D. et al., 2020). Proposed mechanisms of action include: increases in intestinal barrier function (Burger-van Paassen et al., 2009), production of antimicrobials (Jones and Versalovic, 2009), and interaction with the immune system (Veckman et al., 2004; Smits et al., 2005; Kwon et al., 2010). Hence, further studies provide the opportunity to determine mechanistic links between the current generation probiotics and disease, and can inform the production of more targeted therapeutics for a wider range of conditions.

**FIGURE 1:**
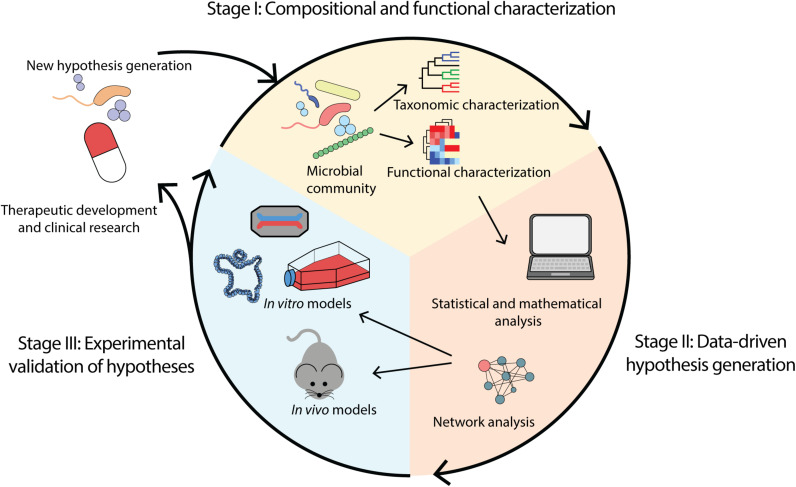
Overview of microbiome analysis workflow for the purpose of determining causality and enabling therapeutic development. (i) Compositional and functional characterization of a microbial community through identification of taxa and functions present. (ii) Data-driven hypothesis generation through application of computational methods including mathematical and statistical modeling. (iii) Experimental validation of hypotheses using *in vivo* and *in vitro* models such as animal models, cell lines, organoids and organ-on-a-chip.

Sequencing and experimental analyses of the microbiome continue to advance substantially. Coupling multi-omics technologies, statistical and computational analyses, and more advanced disease models, these approaches promisingly provide the opportunity to establish disease causality and subsequently inform therapeutic development. We propose a workflow constituent of stages: (i) compositional and functional characterization of the microbiome, (ii) data-driven hypotheses generation, and (iii) experimental validation of hypotheses (Figure 1).

## Compositional and Functional Characterization of the Microbiome (Stage I)

### Taxonomic Composition

As we emerge from the “unculturable” era of microbiome research, it is clear that the application of culture-independent sequencing methods has delivered substantial understandings of the taxonomic composition within bacterial communities. Studies involve experimentally extracting genomic DNA from samples of interest and grouping strains and species based on their genetic similarity in small genomic regions. Reads may be binned based on similarity thresholds, as operational taxonomic units (Westcott and Schloss, 2015), or biological sequences may be statistically distinguished from sequencing errors, as amplicon sequence variants (Eren et al., 2013; Tikhonov et al., 2015; Callahan et al., 2017). The output may be used to describe the community based on taxonomy or phylogeny. These methods have focused on correlating disease with microbial community composition and population structure, allowing the identification of key microbial groups that may mediate disease, yet all have inherent advantages and disadvantages (Table 1).

**TABLE 1:** Advantages and disadvantages of available taxonomic and functional characterization technologies.

Technology	Advantages	Disadvantages
16S rRNA profiling	• Higher sensitivity	• Low taxonomic resolution • PCR amplification bias • Functional characterization dependent on extrapolation
Reference-based metagenomics	• May achieve species and strain level taxonomic assignment for some taxa • Incorporates functional information • No amplification necessary	• Highly dependent on quality and diversity of reference databases • Cannot determine difference between expressed and non-expressed genes
Metagenome-assembled genomes (MAGs)	• No amplification necessary • Identifies species and phylogenies within a sample • Can be used to increase reference catalogs	• Difficulty with high complexity datasets • Difficulty assembling repeat sequences
Multi-omic analysis	• Identifies functional genes, transcripts, proteins and metabolites • Identifies potential mechanisms of action	• Difficulty with data integration • Temporal and spatial sampling concerns

#### 16S rRNA Amplicon Sequencing

Following sample collection, one approach to identify bacterial and archaeal taxonomy is amplification and sequencing of the 16S rRNA gene. Culture-independent, high-throughput, short read sequencing of genetic hypervariable regions, such as with 454 pyrosequencing, Ion Torrent^®^ and Illumina^®^ platforms, provide insight into microbial community composition, and has been beneficial in many fields, such as soil (Schöler et al., 2017) and marine (Wilson et al., 2010) environmental microbiology. The cost-effective nature of 16S rRNA sequencing facilitates studies with extremely large sample sizes (Minich et al., 2018), enabling monitoring of large communities over time (Poretsky et al., 2014), as well as the identification of specific taxa through targeted amplification (Berry and Gutierrez, 2017), or pathogen screening (Watanabe et al., 2018; Numberger et al., 2019). More recently, the application of long-read 16S rRNA gene sequencing has facilitated greater taxonomic resolution than available with amplicon-based 16S rRNA profiling (Johnson et al., 2019; Numberger et al., 2019).

In the gastrointestinal microbiome specifically, 16S rRNA profiling has also been widely applied (Eckburg et al., 2005; Turnbaugh et al., 2007; Gomez-Arango et al., 2016; Li et al., 2020; Ryan et al., 2020). The Human Microbiome Project (HMP) has applied this technology to characterize the complexity of the microbiome at different body sites in over 300 individuals (Turnbaugh et al., 2007), while diet and nutritional associated taxonomic changes to the microbiome have also been described (Zackular et al., 2016; Raman et al., 2019). In disease pathogenesis, substantial progress has also been made in identifying key microbial groups. *Escherichia* and *Faecalibacterium* genera have been associated with IBD disease phenotypes (Frank et al., 2011; Lopez-Siles et al., 2018), while a depletion in butyrate-producing *Coprococcus* spp. has been associated with depression (Valles-Colomer et al., 2019).

Despite the success of 16S rRNA profiling, it may not capture sufficient genomic variation to distinguish between closely related and yet functionally different species (Neville et al., 2018; Johnson et al., 2019). In addition, the reliance on “universal” primers for DNA amplification may introduce biases whereby some species are amplified more than others, with taxonomic coverage reported from 11% to 93% depending on primer choice (Thomas et al., 2012). Other sequencing artifacts, such as polymerase errors (Cline et al., 1996), chimeras (Haas et al., 2011; Eloe-Fadrosh et al., 2016), 16S rRNA copy number variation (Louca et al., 2018), and laboratory contamination (de Goffau et al., 2019; Han et al., 2020) are all exacerbated during PCR amplification.

#### Shotgun Metagenomic Sequencing

As it does not rely on amplification of specific genetic markers, metagenomic shotgun sequencing provides a capacity to measure all genes in the community, thus overcoming many of the limitations of amplicon sequencing. With appropriate sequencing depth and analysis, this approach provides the potential to achieve species and strain level resolution and the foundations for functional characterization (i.e., the metabolic capacity of the microbiome).

Where extensive databases of complete genome sequences exist, reference-based methods of analysis may provide an ability to achieve high-resolution taxonomic classification through direct comparison (Lloyd-Price et al., 2017; Forster et al., 2019). Several programs exist for aligning millions of sequenced reads (or *k-mers*) to reference databases for taxonomic assignment, for example Kraken2 (Wood et al., 2019), MetaPhlAn2 (Truong et al., 2015), Metacache (Müller et al., 2017), CCMetagen (Marcelino et al., 2020a), and Centrifuge (Kim D. et al., 2016). While available metagenomic classifiers each have advantages and limitations that impact accuracy and resolution of classification (McIntyre et al., 2017), reference-guided analysis is also fundamentally limited by availability and selection of the reference genome database. Recent evidence suggests that databases with limited taxonomic diversity also cause misclassification of reads, as they align to evolutionarily conserved regions (Marcelino et al., 2020b). Large scale efforts, such as the Genome Taxonomy Database, which currently contains over 190,000 bacterial reference genomes (Parks et al., 2020), ensure that a comprehensive mix of references are available. Not only does this mitigate misassignment of reads, but it improves taxonomic resolution. In the context of the gut microbiome, Forster et al., improved taxonomic classification by 61% and achieved a subspecies level resolution for 50% of reads by adding 737 bacterial genomes into a reference database of genomes from the HMP collection (Forster et al., 2019).

Where the databases to support reference-based metagenomics are not available, *de novo* assembly of metagenomic sequence reads can be used to obtain metagenome assembled genomes (MAGs; Hugerth et al., 2015; Kang et al., 2015). This can be incredibly effective to identify new species and their phylogenies, as well as to provide catalogs of reference genomes (Parks et al., 2017; Almeida et al., 2019). However, this method can encounter difficulty reassembling repetitive sequence regions (Kingsford et al., 2010) and has problems with incorrect genome reconstructions in the presence of high complexity datasets with genetically similar community members (Sczyrba et al., 2017). Increased sequencing depth and inclusion of hybrid assemblies containing short and long reads or using single cell genomics (Zhang et al., 2006; Chijiiwa et al., 2020) aid in overcoming these issues (Bertrand et al., 2019; Xie et al., 2020); however, this remains cost prohibitive in many circumstances.

Accurate taxonomic classification of metagenomic sequences, relies on quality, diverse and well- populated reference databases. This calls for coordinated cross-disciplinary efforts to build these references using large, diverse datasets such as was undertaken recently for the human gastrointestinal microbiome (Forster et al., 2019; Almeida et al., 2021).

### Functional Characterization

The phenotypic profile of the microbiome community is determined by the functional gene products from the microbiome. Microbial gene products perform several essential processes for human health, including synthesis of vitamins (Hill, 1997), breakdown of non-digestible carbohydrates (Flint et al., 2008), and aid in host immune development (Imaoka et al., 1996; Ivanov et al., 2009). Conversely, microbial gene products also confer virulence in microorganisms, activate inflammatory signaling pathways (Schirmer et al., 2016; Halfvarson et al., 2017) and trigger autoimmune diseases (Wu et al., 2010; Azad et al., 2013). Hence, deleting, altering or introducing various functional genes by manipulating the microbiome may be the key to producing microbiome-based therapies and managing related diseases. There are several techniques to perform functional assessment of the gastrointestinal microbiome, including functional inference from 16S rRNA sequencing, functional prediction from metagenomic sequencing and direct measurement through multi-omics studies (Table 1).

#### Inference of Functional Capacity Based on 16S rRNA Sequencing

Functional capacity may be inferred based on known functions of the closely-related microorganisms identified by 16S rRNA amplicon sequencing (Langille et al., 2013). For example, Stevens et al. (2020), used PICRUSt2, a tool to predict function from 16S sequences (Douglas et al., 2020), to identify butyrate degradation and Gamma-aminobutyric acid degradation pathways in the gut microbiome of individuals with a depression phenotype, contrasted with a prevalence of butyrate producing species in healthy subjects. Predictions from 16S rRNA have also been successful in identifying metabolic shifts between microbiome data sets, as Noecker et al. (2016), identified that metabolites involved in bacterial vaginosis tended to be well predicted by their 16S rRNA prediction model. Functional prediction tools based on marker genes are better at predicting function in relation to different categories. When comparing metagenome prediction tools and metagenomic sequencing results, Sun et al. (2020), highlight that metagenome prediction tools, PICRUSt (Langille et al., 2013), PICRUSt2 (Douglas et al., 2020), and Tax4Fun (Aßhauer et al., 2015) are better at inferring function for “housekeeping” genes involved in translation and transcription, and metabolism-related functions, than for functions related to signaling molecules and signal transduction.

Microbes which exhibit identical 16S rRNA genes show functional similarities, however, there are also important differences which is a limitation of this approach. For example, in *Escherichia coli* approximately 20% of genes are core genes found in all strains. The remaining 80% are accessory genes that are not found in every strain and therefore contribute to the functional diversity within the species (Lukjancenko et al., 2010). As taxonomic resolution of 16S rRNA amplicon sequencing is largely limited to the genus level, inferring function based on taxonomic relatedness at this level is insufficient for comprehensive assessment of functional capacity. Additionally, where reference genomes are not available, no functional inference can be made.

#### Functional Assessment Based on Metagenomic Sequencing

Shotgun metagenomic sequencing provides a direct measurement of all genes present, which can be used to determine all potential functional gene products in the community. To assess functional capacity, sequenced shotgun metagenomic reads are mapped directly to reference gene or genome databases (Kanehisa and Goto, 2000; Consortium, 2020), or assembled into MAGs. Functions can be inferred through conventional gene prediction and annotation workflows such as Prokka (Seemann, 2014), DRAM (Shaffer et al., 2020), and KoFamScan (Aramaki et al., 2020).

Several studies highlight the power of functional prediction in ascertaining the phenotype of a microbial community using metagenomic sequencing. Perturbations of essential microbial functions, including short-chain fatty acid production and L-arginine synthesis, both involved in intestinal barrier function, have been identified in IBD (Vila et al., 2018; Parada Venegas et al., 2019). Additionally, Qin et al. (2012), have characterized microbial functional differences in type 2 diabetes, indicating enrichment in branched-chain amino acid transport, methane metabolism, sulfate reduction, and butyrate synthesis. While the exact roles of these functional changes may be unknown, these studies allow the postulation of potential disease mechanisms that now require statistical analyses and experimental validation.

Unfortunately, a substantial part of the functional capacity within the human microbiome remains unknown. Despite application of advanced computational approaches (Rifaioglu et al., 2019), with an increasing number of hypothetical protein sequences (Nauman et al., 2019), and recent identification of over 4000 small proteins with unknown function in the human microbiome, (Sberro et al., 2019) these limitations remain significant.

#### Multi-Omic Analysis

The requirement for analysis of functional state is not limited to assessing complete genomic capacity, as all genes present within a microbial community may not all be expressed (Turnbaugh et al., 2010; Franzosa et al., 2014, 2018). Therefore, attaining a holistic compositional and functional assessment of the microbiome requires an integrated -omic approach, with metagenomics, metatranscriptomics, metaproteomics, and metabolomics. Metatranscriptomics involves sequencing RNA transcripts from viable microorganisms for insight into gene expression profiles within the community (Moran, 2009), while protein profiles can be identified using metaproteomics (Wilmes and Bond, 2006) and metabolomics can enable the identification of metabolites (Fiehn, 2002). The multi-omics approach provides greater accuracy in determining the role of bacterial strains in disease pathogenesis by capturing the spectrum of genetic potential to phenotype (Heintz-Buschart and Wilmes, 2018).

As with metagenomic approaches, multi-omic data analysis is also largely limited by reference catalogs, generating bias toward previously characterized pathways. Multi-omics datasets generate a multitude of data types (gene content, expression, and microbial species, etc.), and this inherent complexity is challenging to integrate. The different sequencing outputs, such as sequence reads and metabolite counts, are not readily comparable (Palsson and Zengler, 2010; Jiang et al., 2019; Pinu et al., 2019). Differences in rates of transcription, translation and metabolization between different species within a sample call for extensive temporal sampling, which is often not feasible. Additionally, it is important to note that an increase in the number of samples and data types results in high data dimensionality as the number of variables to consider increases (Gloor et al., 2017). Without appropriate statistical correction, biases are introduced into results and conclusions can be drawn from false positives (Thorsen et al., 2016; Gloor et al., 2017). Furthermore, sampling the gut microbiome incurs additional difficulties, as it contains distinct communities and spatial heterogeneity between gastrointestinal regions (Zhang et al., 2014; Lavelle et al., 2015), which are not represented in fecal sampling. Even with extensive sampling, the high dimensionality of multi-omics datasets makes it challenging to determine mechanisms of action. Extensive work is being done in an attempt to overcome these issues *in silico* [see (Meng et al., 2016)].

Despite these challenges, integration of coupled multi-omic datasets from gastrointestinal microbiome samples has been successful in identifying potential links between microbiome functions and disease. Heintz-Buschart et al. (2016), used metaproteomics and metatranscriptomics to correlate the presence of human pancreatic enzymes in type 1 diabetes, with the expression of several microbial pathways, including glycolysis and thiamine synthesis, postulating their role in disease pathogenesis. Similarly, metabolites associated with *Subdoligranulum* species have been correlated with IBD (Lloyd-Price et al., 2019). Hence, the use of multiple dataset types provides the opportunity to link compositional microbial taxa to disease through potential mechanisms of interaction.

## Data-Driven Hypotheses Generation (Stage II)

Taxonomic and functional information are the baseline to derive hypotheses and guide the experiments that can ultimately elucidate causal links between the microbiome and disease and inform therapeutic development. Data- driven hypotheses generation is therefore a key step to achieve translation of microbiome knowledge. Here we outline the most common challenges and emerging approaches in this field.

### Data Processing

Measurements of microbiome datasets are frequently compositional whereby they are proportional and dependent on the analysis approach and sequencing depth (Gloor et al., 2017). As compositionality can strongly influence analysis and conclusions, normalization processes that treat microbiome data as ratios, such as centered log-ratio transformations, must be used before applying standard statistical analysis (Gloor et al., 2016, 2017; Lê Cao et al., 2016).

### Statistical and Machine Learning Methods to Identify Microbiome-Mediated Mechanisms of Health and Disease

Multivariate statistics have been widely used to identify specific bacterial taxa, potentially associated with disease. Discriminatory methods, such as discriminant analyses, aim to define the taxa (or functions) that maximize differences between groups, such as healthy and diseased cohorts. By comparing the gut microbiota of individuals with atherosclerotic cardiovascular disease and healthy controls, Jie et al. (2017), reported a depletion in *Bacteroides* and *Prevotella* and an increase in abundance of *Enterobacteriaceae* and *Streptococcus* spp., identifying potential targets for microbiome modulation. These methods have also been used to identify potential biomarkers, or non-invasive early markers of disease, such as for colorectal cancer including *Fusobacterium nucleatum* and *Parvimonas micra* (Yu et al., 2017), and secondary colorectal metachronous adenoma including *Escherichia* and *Acinetobacter* (Liu Y. et al., 2020). Statistical algorithms that specifically account for compositional microbiome data, such as *selbal* (Rivera-Pinto et al., 2018), can be used to identify groups of taxa that may explain a variable of interest or disease phenotype.

Machine learning has been broadly applied in the context of human microbiome research, in antibiotic resistance prediction and modeling (Arango-Argoty et al., 2018; Rahman et al., 2018), taxonomic classification of metagenomic and 16S rRNA sequences (Vervier et al., 2016; Fiannaca et al., 2018; Desai et al., 2020), and gene function prediction (Cai et al., 2020). However, recent use in identifying microbial signatures and potential therapeutic targets in disease, may inform experimental validation and focus hypothesis generation for disease treatments. These methods have been able to predict incidence of IBD (Hacılar et al., 2018), IBS (Fukui et al., 2020), and colorectal cancer (Thomas et al., 2019) based on microbiome signatures. Notably, these methods can also identify potential mechanisms of disease, for example Thomas et al., have leveraged taxonomic and functional metagenomic information from healthy controls and patients with colorectal cancer, using a random forest classifier to identify reproducible biomarkers. They also reported a potential mechanism of action for colorectal cancer pathogenesis, as gene variants involved in synthesis of trimethylamine from choline in *Hungatella hathewayi* and *Clostridium asparagiforme* were significantly associated with colorectal cancer samples (Thomas et al., 2019). Similarly, Cai et al. (2017), identified microbial pathways, or reaction groups, potentially involved in IBD when analyzing data from Qin et al. (2010), using Non-Negative Matrix Factorization. Both ascorbate and aldarate metabolism, and amino sugar, nucleotide sugar metabolism, fructose and other metabolic pathways were identified to have a greater contribution to IBD samples. While machine learning techniques are promising for microbiome hypothesis generation, there are several challenges including: interpretability (Ghannam and Techtmann, 2021), incomplete data labels (Ten Hoopen et al., 2017), and choosing a model appropriate for the characteristics of the data (Namkung, 2020). Collaborative efforts in machine learning for microbiome analysis, such as that of the European Cooperation in Science and Technology network “ML4Mirobiome” aim to standardize and synergize different fields, and combat these challenges.

### Metabolic Models Enable Mechanistic Predictions About Microbiome Functioning

Genome-scale metabolic reconstructions are based on genomic, biochemical, multi-omics and published experimental data, and can be used to model the response of individual microbial species to changing environmental or dietary conditions and to infer microbial interactions (Thiele and Palsson, 2010; Magnúsdóttir and Thiele, 2018). For example, El-Semman et al. (2014), identified that when *Bifidobacterium adolescentis* was present, *F. praunitzii* changed its metabolism by increasing butyrate production, a short chain fatty acid associated with health. Similarly, Steinway et al. (2015), employed metabolic models to study the ability of different commensal microbes to decrease the abundance of *Clostridioides difficile*, finding that *Barnesiella intestihominis* was able to impede *C. difficile* growth through metabolic interactions. Although experimental validation is required, this knowledge may be used to develop *B. intestinihominis* as a therapeutic target. Metabolic models can also be used to infer the response of microorganisms to potential therapeutic strategies. Promising drug targets for the pathogen *Klebsiella pneumoniae* have been recently identified using metabolic modeling (Cesur et al., 2020), highlighting the potential of computational models to generate testable hypotheses.

### Ecological Interactions and the Emerging Field of Pharmacomicrobiomics

Members of the microbiome function as an ecological community, and leveraging microbial interactions to develop biotherapeutics is a logically promising avenue. Biological interactions can be represented through networks, where systems’ elements, such as microbes and their metabolites, are represented by nodes, and their interactions depicted as edges. These networks may be based on simple correlation analyses such as standard Spearman correlations, or on more sophisticated alternatives such as Bayesian analyses (Jiang et al., 2019). In the human gut microbiome, Dai et al. (2018), used a correlation network analysis to identify the interactions between microbes enriched in colorectal cancer, whereby *Clostridium* species had the highest centralities, or more co-occurrence interactions than is expected by chance, suggesting that they may play a pivotal role in the disease. Additionally, Zhang et al. (2014), provide evidence that *F. praunitzii* and *Bacteroides coprophilus* co-occur less than is expected by chance, suggesting competition between these two species. Interestingly, competitive exclusion of *F. praunitzii* is associated with inflammation in patients with Crohn’s disease, and therefore *B. coprophilus* has been identified as a potential therapeutic candidate to be targeted for removal from the community (Zhang et al., 2014). Network theory can also be applied to integrated multi-omic data to identify the functional mechanisms behind microbial crosstalk (Lloyd-Price et al., 2019), providing targeted avenues for downstream characterization and validation in experimental models.

The marked influence of the individual genetic background on their response to drugs has been well established in the pharmacogenetics field. More recently, the emerging pharmacomicrobiomics field addresses the many ways in which the microbiome can modulate the host metabolic response to improve the efficacy of therapeutic strategies. For example, it has been observed that cardiac drugs may be inactivated by *Eggerthella lenta* in the gastrointestinal tract (Haiser et al., 2013). Additionally, in several forms of cancer, studies have shown that the microbiome can influence patient responsiveness to chemotherapy and immunotherapy [see (Helmink et al., 2019) for a review]. The exact mechanisms by which the microbiota influence therapeutic responses are still poorly characterized, but we expect that the ecological interactions among members of the microbiome and their interactions with the host immune system play a fundamental role in various microenvironments. Microbial community assembly and metabolism is known to be context-dependent, with particular pathways switching on and off depending on their biotic and abiotic surroundings. Therefore, a systems-biology approach that considers how the diverse microbiome influences host key pro-inflammatory and anti-inflammatory mediators is likely to reveal the most promising personalized therapies.

## Experimental Validation of Hypotheses (Stage III)

While statistical and computational analyses are critical in the identification of patterns in microbiome function and interactions, it is important to test these hypotheses experimentally in order to demonstrate causation and translate such findings for clinical application. Research findings that are both computationally and experimentally supported are more likely to render positive results at the clinal trial stage. Various tools and technologies have emerged for experimental hypotheses validation which encompasses both *in vitro* and *in vivo* approaches.

### *In vivo* Models

Mouse models can provide insights into host-microbial interactions, including how the microbiota shapes host immunity (Maier and Hentges, 1972; Bouskra et al., 2008), physiology, and metabolism (Cash et al., 2006). There are four main mouse models used in microbiome studies; specific pathogen-free (SPF) mice, bred for the absence of murine disease-causing pathogens (e.g., *Helicobacter pylori*)*;* antibiotic treated (abx) mice, which are depleted of all or specific microbial groups; germ free (GF) mice are absent of any microbes; and gnotobiotic mice, which are selectively recolonized with either single microbes or defined communities. Germ free and gnotobiotic mice have the advantage of a controlled microbiome composition; however, require specific maintenance and breeding strategies to ensure appropriate development of lymphoid tissue (Bouskra et al., 2008) and mediation of immune response (Levy et al., 2015). In contrast, SPF and abx mice undergo normal immune development but lack a defined microbial community.

Specific pathogen-free mice have been utilized to elucidate the potential impact of the microbiome composition on host immune regulation and disease pathogenesis (Vijay-Kumar et al., 2007; Letran et al., 2011). In contrast to SPF mice, abx mice are administered with narrow spectrum antibiotics, hence rendering them more susceptible to infection due to the absence of the gut microbiome. Various studies have thus utilized the abx model to affirm the necessity of the gut microbiome in pathogen colonization resistance and immune response to infection (Abt et al., 2012; Deshmukh et al., 2014).

Germ free mice can be crucial to demonstrate the transmissibility of the microbiome and associated phenotype through fecal microbiome transplantation from donor to recipient animals (De Palma et al., 2017; Zhou et al., 2017; Le Bastard et al., 2018), and this model has led to the development of humanized gnotobiotic mice (Hazenberg et al., 1981; Turnbaugh et al., 2009; Chung et al., 2012). Generation of gnotobiotic mice from “diseased” or “healthy” human samples, with optimized diet and environmental exposures, may better reflect a human system (Park and Im, 2020).

Despite these important insights, the highly co-evolved nature of microbe and host (Ochman et al., 2010; Goodrich et al., 2014) introduce difficulties for human translation. Microbe species differ greatly between humans and mice, with many human-derived microorganisms failing to colonize mice (Kibe et al., 2005; Chung et al., 2012). For example, *Lactobacillus reuteri* has been identified in both human and mouse gastrointestinal tracts (Frese et al., 2011). However, strains present in rodents have been found to contain rodent specific genes that impact the colonization and function of the species in the murine model (Frese et al., 2011). Additionally, it has been shown that commensal (Lasaro et al., 2014) and enterohaemorrhagic strains (Roxas et al., 2010) of human *E. coli* are unable to permanently colonize conventionally reared mice. Therefore, despite the invaluable immunological insights obtained from animal *in vivo* models, their lack of congruency with the human microbial community structure and immune function (Chung et al., 2012; Eun et al., 2014), suggests the need for human-specific models in microbiome disease research.

### *In vitro* Models

#### Immortalized Cell Lines

Human-derived immortalized cell lines are a common tool to gain a better understanding of host-microbial functions due to several advantages (Bahrami et al., 2011; Sadabad et al., 2015). Immortalized cell lines are cost-effective, can continuously divide and proliferate, providing a means for efficient high-throughput and reproducible analyses. Human-derived cell lines widely utilized in the gastrointestinal field include intestinal epithelial-derived IEC-6 cells and the colonic adenocarcinoma-derived T84, HT-29, LS513, and Caco2 cell lines, which have been used to demonstrate the beneficial impact of probiotics, including strains of *Lactobacilli* and *Bifidobacteria* in enteroinvasive *E. coli* invasion (Khodaii et al., 2017). However, epithelial cell lines vary in gene expression compared to the normal intestinal epithelium within the human body (Bourgine et al., 2012) and, being comprised of a single cell type, are absent of endothelial and immune cell populations, which limits the ability to biologically represent human gut physiology and morphology (Pearce et al., 2018). Additionally, a monolayer of cells grown in a flask or petri dish cannot recapitulate the anaerobic conditions and epithelial structure present in the intestinal lumen which is the ecological niche of a large proportion of the microbiota. Therefore, it is difficult to effectively capture the native state of microbe and host using immortalized cell lines. While these models provide fundamental information on host cellular response to microbes, there remains a need for models capable of capturing the cellular diversity and anaerobic intestinal environment in order to appropriately assess host-microbe interactions.

#### Organoids

*In vitro* intestinal organoids, referred to as “mini-guts” or enteroids/colonoids, are derived from intestinal stem cells and are self-organized into an enclosed, three-dimensional structure with a heterogeneous array of organ specific cell types (Sato et al., 2009). Derivation of organoid tissue models and co-culture methods have been well established and utilized in drug-screening, investigations of gastrointestinal infections, host-pathogen and host-microbiome interactions (Finkbeiner et al., 2012; Forbester et al., 2015; Hou et al., 2018; Driehuis et al., 2020). Additionally, *ex vivo* patient-derived organoid cultures (primary cells from human tissues) may be used as a tool for personalized therapeutic treatments (van de Wetering et al., 2015). Microinjection allows the delivery of microbes and substances into the organoid lumen, which has been particularly useful for studies of the microbiome (Williamson et al., 2018). For example, Leslie et al. (2015), used microinjection to demonstrate the loss of epithelial barrier integrity, through injection of viable *C. difficile*, as well as the *C. difficile* exotoxin, toxin A (TcdA). Furthermore, human fecal microbiota can be transplanted and maintained in culture within the intestinal lumen (Williamson et al., 2018). Although organoids can offer insight into disease pathogenesis and host-microbial relationships, organoid systems lack the critical physiological processes (peristalsis, oxygen gradient) and immune-microbiome functions (vasculature, intestinal immune cells) that are necessary to advance our understanding of the microbiome. As these are important factors when investigating human gut physiology and function, the limitations of organoid models may reduce the ability to decipher the complexity of host-microbiome crosstalk. Thus, it is essential to consider alternative experimental models that are more biologically accurate.

#### Organ-on-a-Chip

Organ-on-a-chip technology has revolutionized the ability to recapitulate organ-level physiology. The lung-on-a-chip was the pioneering platform that successfully micro-engineered the lung-alveoli vasculature and function, including controlled breathing motions by applying fluid shear stress on the device (Huh et al., 2010). Similarly, flow-induced motions have been reproduced in gut-chip systems to mimic intestinal peristalsis (Beaurivage et al., 2019). The gut-on-a-chip device models the structure, function, immune capability and other physiological processes in the human gut, all controlled by microfluidics, which allows fine-tuning of fluids at microscale levels (Vickerman et al., 2008; Bhatia and Ingber, 2014). This emerging technology provides a means of hypotheses validation and demonstration of causation in microbiome studies. The gut-on-a-chip device consists of upper and lower microfluidic channels separated by a polymeric porous membrane which allow continued perfusion, mimicking the dynamics of the gastrointestinal system, unlike other existing *in vitro* model systems (Bhatia and Ingber, 2014). The upper channel or apical membrane allows intestinal epithelial cells to be cultured, while media containing cellular growth factors can be perfused through the channel. Endothelial and immune cells (e.g., myeloid cells, leukocytes) can be compartmentalized in the lower channel or basolateral membrane to mimic the intestinal microvasculature, allowing for epithelial-endothelial interactions to occur. For comprehensive analyses of host-microbial pathophysiology, innate and adaptive immune responses can be selectively modeled by differentiating patient-derived primary cells into various immune cell subsets (e.g., macrophages, dendritic cells; Maurer et al., 2019). In addition, these systems can maintain an oxygen gradient from the aerobic microvasculature channels to the anaerobic epithelial layer, allowing for co-culture of a diverse array of aerobic and anaerobic gastrointestinal microbes (Jalili-Firoozinezhad et al., 2019). A microbiome can therefore be established at a tissue-interface, and host-microbe responses can be measured in real-time.

Proof of concept studies have demonstrated the efficacy of generating a diverse microbiome *in situ* (Jalili-Firoozinezhad et al., 2019). Specifically, Kim H.J. et al. (2016), demonstrated the role of intestinal probiotic bacterial strains in modulating inflammation by enhancing intestinal barrier function. Similarly, this technology has been used to model the attachment, invasion and propagation of *Shigella* in the intestinal epithelium (Grassart et al., 2019) and the involvement of commensal microbial metabolites in colonic epithelium injury during enterohemorrhagic *E. coli* infection (Tovaglieri et al., 2019). In addition to modeling host-bacterial interactions, organ-on-a-chip models can also be used for identification and testing of therapeutic bacterial candidates. To target specific health and disease states, patient-derived primary cells can be isolated from biopsies and then cultured in gut-chip devices to fine-tune analysis of host response toward drugs, probiotics or pathogens (Kasendra et al., 2018; Van Den Berg et al., 2019). The organ-on-a-chip is an invaluable tool for studying the role of the microbiome in health and disease in various tissue types of the human body. The scalable nature of this technology provides a means to assemble different organ-chips to produce a human-chip system for investigations on an inter-systemic level (Maschmeyer et al., 2015; Picollet-D’hahan et al., 2021). It will also provide significant insight of the interconnectivity between the gut microbiome with other tissue or organ systems. While further research and development is underway, an established organ-on-a-chip platform could enhance the ability to validate hypotheses in a high-throughput manner and pave way for the development of therapeutics toward efficacious personalized precision medicine.

## Concluding Remarks

Human gastrointestinal microbiome research has the potential to deliver critical clinical and therapeutic development if it shifts toward mechanistic studies. We proposed that a suitable avenue to translate microbiome research is a workflow that includes (i) compositional and functional characterization of the microbiome, (ii) data-driven hypotheses generation, and (iii) experimental validation of hypotheses. By integrating recent advances in computational, statistical and experimental methods through this workflow, the ability to identify disease causation and propose logical microbiome therapeutics in disease treatment is possible.

The next step toward implementation of microbiome-based treatments relies on a deeper biological understanding of how microbial communities respond to the introduction of new strains or microbial mimetics. As such, research into ecological interactions and pharmacomicrobiomics will be key to understand the complex ways in which the microbiome influence host health and response to drugs. We expect that a systems-biology approach targeting the microbiome and host-microbiome interactions will guide the successful implementation of microbiome therapeutics.

## Author Contributions

RBY and SCF conceptualized the idea. RBY wrote the manuscript and made the figure. VRM, MC, ELG, and SCF contributed to various sections and edited the review. All authors contributed to the article and approved the submitted version.

## Conflict of Interest

SCF is a consultant to BiomeBank, Australia. The remaining authors declare that the research was conducted in the absence of any commercial or financial relationships that could be construed as a potential conflict of interest.
